# Adsorption Profile of Basic Dye onto Novel Fabricated Carboxylated Functionalized Co-Polymer Nanofibers

**DOI:** 10.3390/polym8050177

**Published:** 2016-04-29

**Authors:** Marwa F. Elkady, Mohamed R. El-Aassar, Hassan Shokry Hassan

**Affiliations:** 1 Chemical and Petrochemical Engineering Department, Egypt-Japan University of Science and Technology, New Borg El-Arab City, Alexandria 21934, Egypt; 2 Fabrication Technology Department, Advanced Technology and New Materials Researches Institute, City of Scientific Researches and Technological Applications, New Borg El-Arab City, Alexandria 21934, Egypt; 3 Polymer Materials Researches Department, Advanced Technology and New Materials Researches Institute, City of Scientific Researches and Technological Applications, New Borg El-Arab City, Alexandria 21934, Egypt; mohamed_elaassar@yahoo.com; 4 Electronic Materials Researches Department, Advanced Technology and New Materials Researches Institute, City of Scientific Researches and Technological Applications, New Borg El-Arab City, Alexandria 21934, Egypt; hassan.shokry@gmail.com

**Keywords:** nanofiber functionalization, electrospun nanofiber, dye removals, equilibrium and kinetic modeling

## Abstract

Acrylonitrile-Styrene co-polymer was prepared by solution polymerization and fabricated into nanofibers using the electrospinning technique. The nanofiber polarization was enhanced through its surface functionalization with carboxylic acid groups by simple chemical modification. The carboxylic groups’ presence was dedicated using the FT-IR technique. SEM showed that the nanofiber attains a uniform and porous structure. The equilibrium and kinetic behaviors of basic violet 14 dye sorption onto the nanofibers were examined. Both Langmuir and Temkin models are capable of expressing the dye sorption process at equilibrium. The intraparticle diffusion and Boyd kinetic models specified that the intraparticle diffusion step was the main decolorization rate controlling the process.

## 1. Introduction

Recently, the synthesis, modification and functionalization of polymeric materials using different techniques have represented the main target for scientists, especially as they focus their research in fields related to water and wastewater treatment processes. Accordingly, there are a large number of synthetic and natural polymeric materials that are characterized by their functional groups suitable for dye decolorization processes [1,2]. Polyacrylonitrile (PAN) is characterized by its unique and well-known properties, so it is considered as one of the most important precursors for polymer fabrication materials. Polyacrylonitrile-based polymeric materials are distinguished by their hardness, chemical stability and compatibility with polar substances [3]. So, they can be easily chemically functionalized with pendant cyano groups to attain novel adsorbent polymeric material characterized by its amidoxime (AO) group that gives the polymeric material new properties to be suitable for different applications such as waste water purification and metal chelation [4]. Poly(acrylonitrile-*co*-styrene), which is an important random copolymer of styrene and acrylonitrile, has excellent resistance for chemicals and oil; it also possesses high rigidity, good heat resistance and superior transparency, and is suitable for different applications such as immobilization of catalytic materials, harmful chemical filtration, and packaging and medical applications [5]. These superior properties of poly(acrylonitrile-*co*-styrene) demonstrate that the co-polymer may be utilized as an efficient adsorbent material for harmful pollutant decontamination from wastewater.

The discharge of polluted waste streams contaminated with coloring or dying substances into the environmental water affects mainly the aquatic life. These pollutants retard the photosynthesis process which inhibits the growth of aquatic biota through obscuring the sunlight and consuming the dissolved oxygen [6]. Some dyes may cause allergic dermatitis, skin irritation, cancer and mutations in man. There are several types and classifications of dyes such as basic, acid, azo, reactive, cationic and anionic dyes [7]. Basic violet 14 (BV 14) is a cationic dye, highly water soluble and nonvolatile. This dye type is utilized mainly in the leather and textile industries and in paints and inks [8]. BV 14 directly harms skin, eyes, and the gastrointestinal respiratory tract. Moreover, this dye may provoke phototoxic and photo allergic reactions [9]. This type of dye was characterized by its carcinogenic toxicity in both humans and animals [10]. There are many methods used to remove dye contaminates from polluted water; however, most of the known decolorization techniques may be not efficient for complete dye decolorization or may require expensive equipment or consume high energy in their operation [11]. The adsorption technique represents the most economical technique for pollutant decontamination from wastewater. Different nanomaterials have been widely used as adsorbent materials for pollutant removal from aqueous solutions due to their efficiencies. However, most of these nanomaterials suffer from aggregation tendencies due to their hydrophobic nature. These aggregates of nanomaterials reduce the material adsorption capacity and slow down the kinetics of the treatment process. The material adsorption capacity could be enhanced by removing this aggregation phenomenon. Moreover, with respect to the one-dimensional (1D) nanoscale materials, such as nanofibers, they have enhanced properties of the corresponding materials that improve their application potential in diverse areas [12,13]. The polymeric nanofibers possess desirable and superior characteristics, which is due to their greater surface-to-volume ratios. Electrospinning is one of the improved and economical methods to produce polymeric nanofibers with diameters from tens of nanometers to submicrometers. Accordingly, the synthesized poly(AN-*co*-ST) will be fabricated into nanofibers using the electrospinning technique. As an attempt to improve the polarity of the synthesized poly(AN-*co*-ST) nanofibers, it was hydrolyzed to be functionalized with carboxylic functional groups to increase its sorption affinity for cationic contaminates such as dyes and heavy metals during the water purification process [14].

This investigation deals with the fabrication of innovative carboxylated poly(AN-*co*-ST) nanofibers to be utilized for basic violet 14 (BV 14) sorption from polluted wastewater. This work represents the first fabrication of the carboxylated poly(AN-*co*-ST) nanofibers (to the extent of our knowledge). The previous research works deal with the preparation of poly(AN-*co*-ST) nanofibers. The dye sorption performance of the fabricated innovative nanofibers was examined as a function of the contact time and the dye concentration. The equilibrium and kinetics of the dye sorption onto the fabricated nanofibers were established and modeled using different mathematical modeling equations to describe the behavior of the dye sorption process.

## 2. Materials and Methods

### 2.1. Materials

**Figure 1:**
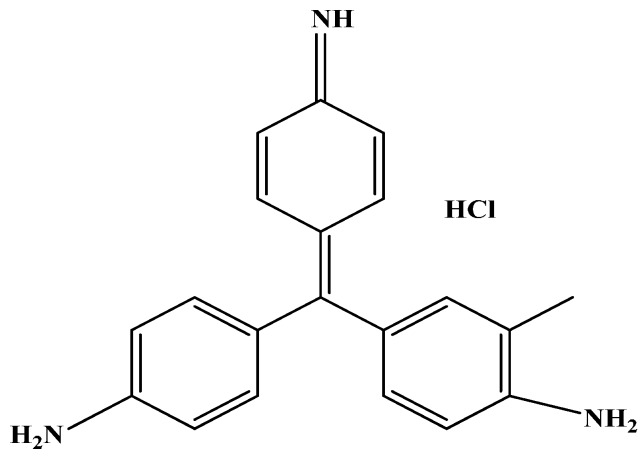
Chemical structure of basic violet 14 dye.

All chemicals utilized for preparation are from analytical grades. Styrene (ST) was supplied from Acros (New Jersey, YSA). Potassium persulfate (K_2_S_2_O_8_) and acrylonitrile (AN) were purchased from BDH (London, UK). Tetrahydrofuran (THF) was derivable from Fluka AG (Buchs, Switzerland). The nominated dye pollutants utilized as adsorbate was the basic violet (C.I. 14, purity 99%) that was provided by Ciba Specialty Chemicals Inc. (Basel, Switzerland). Figure 1 investigates the molecular structure of the basic dye. A stock solution of 1000 mg/L was prepared by dye dissolving in distilled water to prevent and minimize possible interferences; the desired concentrations were achieved through stock dilutions.

### 2.2. Methods

Three different steps should subsequently be followed to fabricate the carboxylated poly(AN-*co*-ST) copolymer nanofiber. Firstly, preparation of poly(AN-*co*-ST) copolymer powder material. The prepared powder copolymer was fabricated into nanofiber using electrospinning technique. Finally this fabricated nanofiber was chemically modified to be functionalized with carboxylic groups.

#### 2.2.1. Synthesis of Poly(AN-*co*-ST) co-Polymer Nanofiber

Firstly, particles from poly(AN-*co*-ST) copolymer were prepared using solution polymerization. The copolymerization process was taken place through mixing the water: ethanol co-solvents with mixing ratio equal to 70:30 in presence of 0.01 M K_2_S_2_O_8_ as initiator at the room temperature. Aniline and styrene monomers were added with equimolar ratios of 1:1 to the previous initiator solution. The polymerization process was carried out at the heated water batch with temperature 55 °C for 4 h. After completing the polymerization process, the polymer particles was separated through centrifugation and washed several times with distilled water to remove any un-reacted monomers or excess initiator. The white polymeric particles were dried at 55 °C overnight. The produced poly(AN-*co*-ST) copolymer was dissolved at 10% THF to attain dissolved polymeric solution suitable for electrospinning. The copolymer solution was fed into the electrospinning (Elmarco s.r.o., Liberec, Czech Republic) collector. The optimum utilized electrospinning parameters for poly(AN-*co*-MMA) copolymer nanofiber fabrication were 5.3 rpm drum speed using 62.3 kV at 17 mm electro-distance with 32% humidity and 22 °C temperature. After finishing the electrospinning process, a homogeneous poly(AN-*co*-MMA) copolymer nanofiber was fabricated at the grounded drum of the electrospinning machine.

#### 2.2.2. Fabrication of Carboxylated Poly(AN-*co*-ST) co-Polymer Nanofiber

The alkaline treatment of the poly acrylonitrile-based polymeric material is designed simply upon the conversion of nitrile groups (C≡N) present at the poly acrylonitrile structure into carboxylic acid groups (–COOH) that is known as carboxylation process. In this regard, poly(AN-*co*-ST) copolymer nanofiber with 25 cm^2^ surface area was immersed at 100 mL of 15% NaOH solution for 1 h at 80 °C. After 1 h, the nanofiber was washed several times with deionized water. Finally, the carboxylated electrospun poly(AN-*co*-ST) nanofibers was treated with 0.1 M HCl and dried at 60 °C for 120 min.

#### 2.2.3. Characterization of Poly(AN-*co*-ST) Electrospun Nanofibers

In order to accomplish the modification at the physicochemical properties of the carboxylated functionalized poly(AN-*co*-ST) nanofiber compared with its parent poly(AN-*co*-ST) nanofiber, both the FTIR and SEM characterization techniques were examined. The presence of the carboxylic group at the functionalized nanofiber was confirmed using FT-IR spectrometer (Bruker, Bremen, Germany). The morphological modification of the functionalized nanofiber was tested using scanning electron microscopy (Joel JSM-6380 LA, Tokyo, Japan). Firstly, the sample surface was gold-sputtered to be examined by SEM. The average diameter of the electrospun nanofibers was determined using scanning electron microscopy software.

#### 2.2.4. Decolorization Process of Basic Violet 14 Dye onto Carboxylated Poly(AN-*co*-ST) Nanofibers

The amount of 50 mL of dye solution of pH = 6.2 with identified concentration was taken in a glass-stopper flask with 0.1 g from the fabricated nanofibers and mixed using thermostatic shaker bath for 60 min. The residual dye solution was analyzed using a UV–Vis spectrophotometer (Labomend. Inc., Los Angeles, CA, USA) at the maximum wavelength (max = 545 nm) via standard calibration curve. The removal efficiency of dye onto the fabricated nanofiber was estimated using the following equation:

Removal (%) = ((*C*_o_ − *C*)/*C*_o_) × 100 (1)

where *C*_o_ is the initial BV 14 concentration (mg/L), *C_t_* is the concentration of BV 14 at specific time (mg/L).

#### 2.2.5. Equilibrium Sorption of Dye onto Fabricated Functionalized Nanofibers

The effect of dye solution concentration was monitored in a batch mode of operation at the adsorption equilibrium time of 30 min. The equilibrium decolorization data were modeled using the linear forms of Langmuir, Frendlich and Temkin equilibrium isotherm equations.

#### 2.2.6. Kinetic Sorption of Dye onto Fabricated Functionalized Nanofibers

The influence of contact time on the BV 14 dye adsorption was monitored using 50 mL of specified initial dye concentration solution (1–50 ppm) that was mixed with 0.1 g of fabricated nanofiber at 400 rpm for different time intervals. After a fixed time interval, 5 mL dye solution samples were drawn and analyzed using the spectrophotometer. The experimental results were tested using pseudo-first-order, pseudo-second-order, and Boyd and intra-particle diffusion kinetic models to explain the dye decolorization kinetic process.

## 3. Results and Discussion

### 3.1. Characteristics Properties of Chemically Modified Fabricated Nanofibers

**Figure 2:**
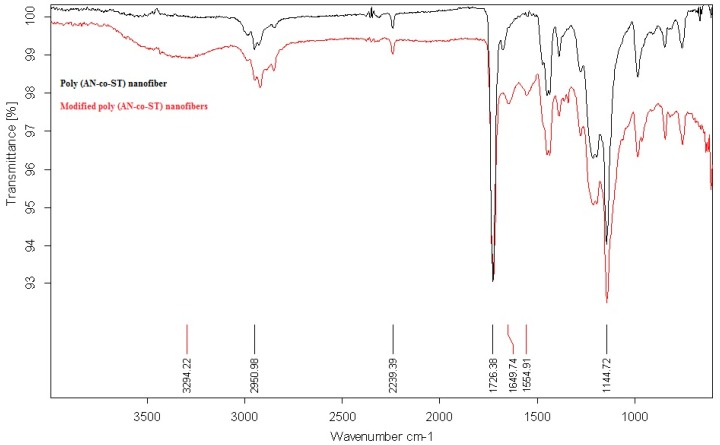
FT-IR spectra of poly(AN-*co*-ST) nanofiber and modified poly(AN-*co*-ST) nanofibers.

The FTIR spectrums of the two fabricated nanofibers before and after the chemical modification process were compared in Figure 2. It was indicated that the spectrum of the non-chemically modified poly(AN-*co*-ST) nanofibers attains main characteristic peaks at 2950 and 2239 cm^−1^ which may be due to the stretching vibration of methylene (–CH_2_–) and nitrile groups (–CN–), respectively. However, the chemically modified nanofibers have the characteristic peak of the carbonyl functional group at around 3294 cm^−^^1^ due to the CO stretching vibration which confirmed the conversion of the –CN– groups into –COOH as a result of the chemical modification process.

**Figure 3:**
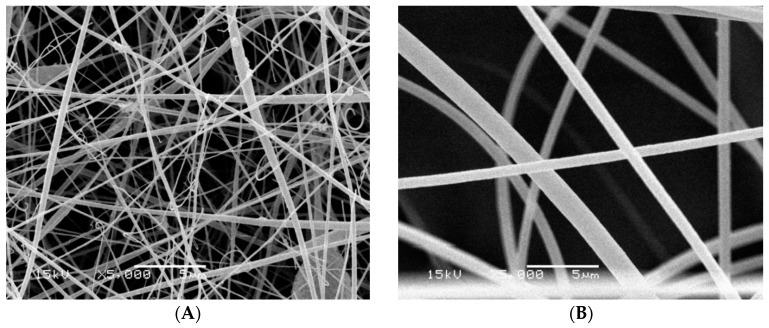
SEM micrographs of (**A**) poly(AN-*co*-ST) nanofibers; and (**B**) modified poly(AN-*co*-ST) nanofibers.

The differences at the morphological structure of the fabricated nanofiber before and after the chemical modification process were detected using scanning electron microscopy (SEM). It was indicated from Figure 3 that the nanofiber morphological structure changed from smooth nanofibers for the non-functionalized nanofibers into a uniform shape structure for the carboxylated nanofibers. The average diameters of modified poly(AN-*co*-ST) nanofibers (370 ± 85 nm) (Figure 3B) were a little bigger than those of neat poly(AN-*co*-ST) nanofibers (260 ± 98 nm) (Figure 3A). It can be explained by the interaction and hydrolysis between NaOH and the copolymer which consequently affects the fiber properties. Increasing the NaOH% concentration changed the morphology of the polymer nanofibers and then the diameter may be due to the increase in the hydrolysis ratio. Also, a more uniform and porous fiber structure was obtained when increasing the percentage of the NaOH.

### 3.2. Decolorization Process of Basic Violet 14 Dye onto Carboxylated Poly(An-co-St) Nanofibers

#### 3.2.1. Equilibrium Sorption of Dye onto Fabricated Functionalized Nanofibers

**Figure 4:**
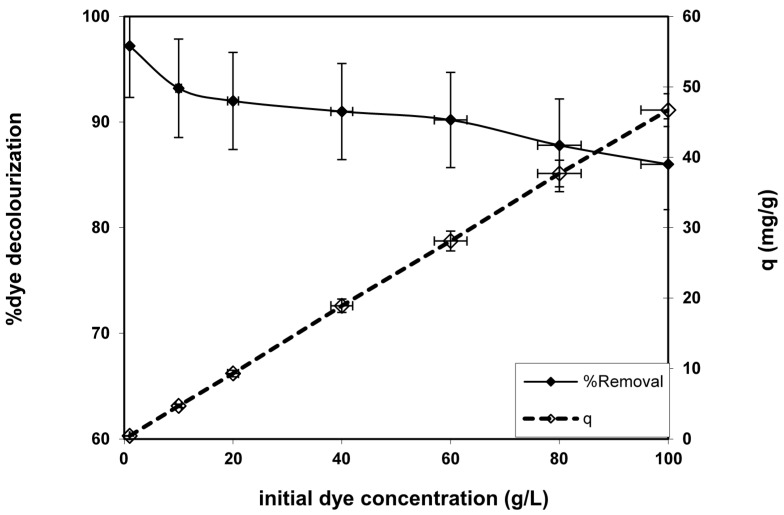
Effect of initial dye concentration on dye removal capacity and percentage decolorization onto the chemically modified nanofiber. (Volume of dye solution = 50 mL, agitation speed = 400 rpm, dosage of nanofiber = 0.1 g, pH = 6.2 and temperature = 25 °C).

The influence of the initial dye concentration variation at the equilibrium at 30 min on both the percentage of dye removal and the amount of dye sorption was investigated in Figure 4. It was indicated that the increment at the initial dye concentration enhances the amount of dye adsorbed onto the fabricated nanofibers. This may be due to the improvement of the mass gradient and the number of dye particle collisions that raise the driving force to overcome all resistances of the dye mass transfer between the liquid and solid phases as a result of initial BV 14 concentration increase. However, the improvement of the initial dye concentration declines with the percentage of dye sorption onto the fabricated nanofibers. The same results were recorded for various dye adsorption processes onto other studied adsorbent materials [15,16].

#### 3.2.2. Isotherm Analysis of Dye Sorption Process

In order to describe the relation between the distribution of the adsorbed molecules between the liquid and the solid phases at the equilibrium state, the equilibrium adsorption isotherms were examined. Accordingly, the equilibrium data for BV 14 dye adsorption onto the fabricated nanofibers were modeled using three adsorption isotherm equations, namely Langmuir, Freundlich and Temkin.

**Figure 5:**
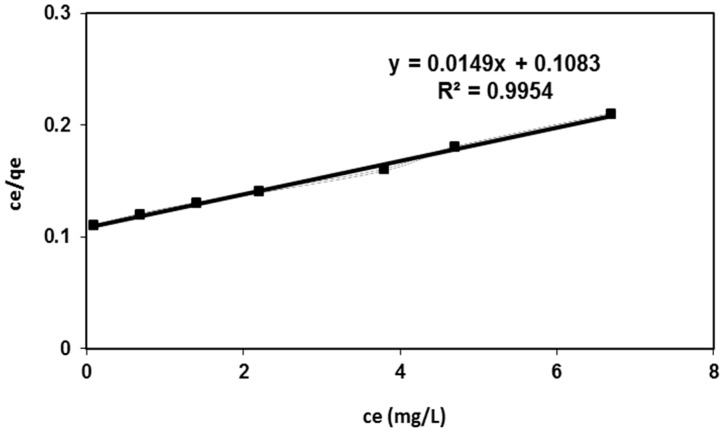
Langmuir adsorption isotherm for BV 14 dye adsorption onto the chemically modified nanofibers.

The adsorption behavior of various adsorbent materials at equilibrium was investigated using the Langmuir isotherm model. The Langmuir equation suggested that the adsorption process takes place onto the adsorbent material through specific homogeneous sites and as the adsorbate molecules occupied the available sites, no adsorption occurs at the adsorbent material [17]. This model may be expressed in linear form as the following:

(2) $$\frac{C_{e}}{q_{e}}\;=\;\frac{1}{q_{m}K}\;+\;\frac{C_{e}}{q_{m}}$$

where *q*_e_ is the amount adsorbed onto the nanofibers (mg/g), *C*_e_ is the equilibrium concentration of dye ions (mg/L), *q*_m_ is the constant that represents the maximum adsorption capacity (mg/g) and *K* is the Langmuir constant related to adsorption energy (L/mg). The linear plot of *C*_e_/*q*_e_ *vs.* *C*_e_ was investigated in Figure 5. This figure indicates that the Langmuir equation fitted well the experimental dye sorption data onto the fabricated nanofibers with an extremely high value of the correlation coefficient (*R*^2^ > 0.99). Accordingly, the Langmuir model represents an accurate description of the dye sorption process.

The dimensionless equilibrium parameter (*R*_L_) that represents the essential Langmuir isotherm characteristics may be expressed as the following:

(3) $$R_{L}=\frac{1}{1\;+KC_{0}}$$

where *K* is the Langmuir constant and *C*_0_ is the initial BV 14 dye concentration. The calculated values of *R*_L_ at different concentrations were found to be in the range of 0–1 (Table 1). This result confirms that the Langmuir isotherm model was appropriate for the dye adsorption process onto the fabricated nanofibers [18]. Hence, the monolayer adsorption process of BV 14 dye onto the fabricated nanofibers was verified.

**Figure 6:**
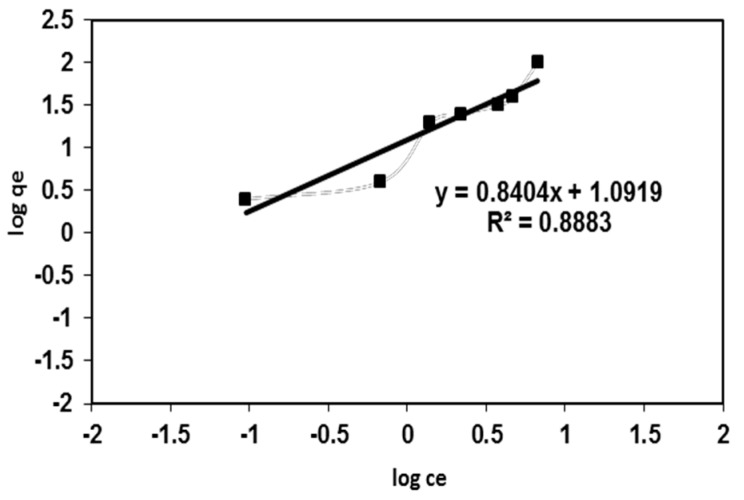
Freundlich adsorption isotherm for BV 14 dye adsorption onto the chemically modified nanofibers.

The Freundlich isotherm model proposes heterogeneous adsorptive energies on the surface of the adsorbent materials, and the linear equation may be written as [19]:

(4) $$\text{ln}\;q_{e}=\text{ln}\;K_{F}+\frac{1}{n_{f}}\ln C_{e}$$

where *K*_F_ is the Freundlich constant related to the adsorption capacity and *n* represents the adsorption intensity. The Freundlich adsorption isotherm of BV 14 onto the fabricated nanofibers was illustrated in Figure 6. It can be seen that the regression correlation coefficients (*R*^2^ = 0.866) for the Freundlich fitting are lower than that for the Langmuir fitting [20]. This result showed that the Langmuir equation gave an accurate description of the experimental data compared with the Freundlich equation. Moreover, the calculated *n* value of the Freundlich isotherm model (Table 1) is greater than 1, given the prediction about the chemisorption nature of the dye adsorption onto the fabricated nanofiber [21].

**Figure 7:**
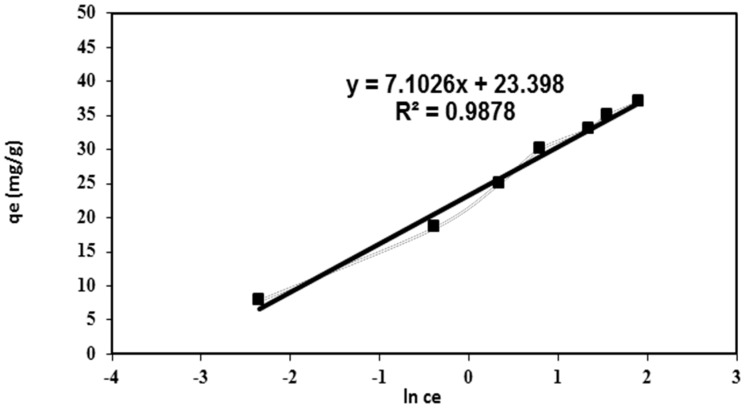
Temkin adsorption isotherm for BV 14 dye adsorption onto the chemically modified nanofibers.

The Temkin isotherm model supposes an interaction between the adsorbate molecules and the adsorbent material. This model suggested that the generated heat of adsorption of all molecules on the layer due to the adsorbate-adsorbent interaction was decreased linearly instead of logarithmically with coverage [22]. The Temkin equation can be written as:

*q_e_* = *B* × ln(*K_T_*) + *B* × ln(*C_e_*) (5)

where *B* is the Temkin constant which is related to the adsorption heat and *K_T_* is correlated to the maximum binding energy. Figure 7 investigates the plots of the Temkin isotherm which consider that the chemisorption interaction of the adsorbate-adsorbent had fitted well with the correlation coefficients (*R*^2^ = 0.988) [21]. This result further supports the chemisorption mechanism for the adsorption of BV 14 onto the fabricated nanofibers.

#### 3.2.3. Kinetic Modeling of Dye Sorption Process onto Fabricated Functionalized Nanofibers

In order to know the detailed mechanism of any adsorption process, the process kinetics should be monitored. So, the experimental data of the kinetic sorption of cationic BV 14 dye onto the chemically modified nanofibers will be tested using pseudo-first-order, pseudo-second-order, intra-particle diffusion and Boyd equations.

**Figure 8:**
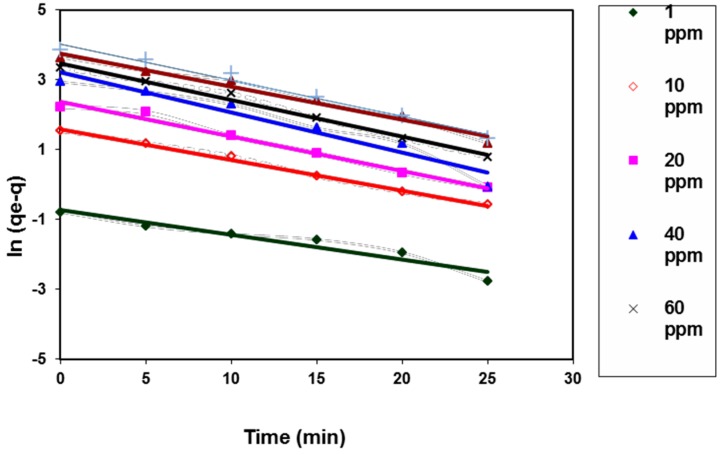
Pseudo-first-order kinetic model for BV14 dye adsorption onto the chemically modified nanofibers.

The widely used pseudo-first-order reaction equation for the liquid–solid adsorption system may be expressed as [22]:

ln(*q*_e_ − *q_t_*) = ln(*q*_e_)− *k*_1_*t* (6)

where *q*_e_ is the adsorption capacity at equilibrium (mg/g), *q_t_* is the adsorption capacity at time *t* (min), and *k*_1_ (min^−1^) is the rate constant of the adsorption process. Figure 8 illustrated the plotting of ln(*q*_e_ − *q_t_*) *vs.* time. The calculated constants from the slope and intercept of the linear plotting were tabulated in Table 2.

**Figure 9:**
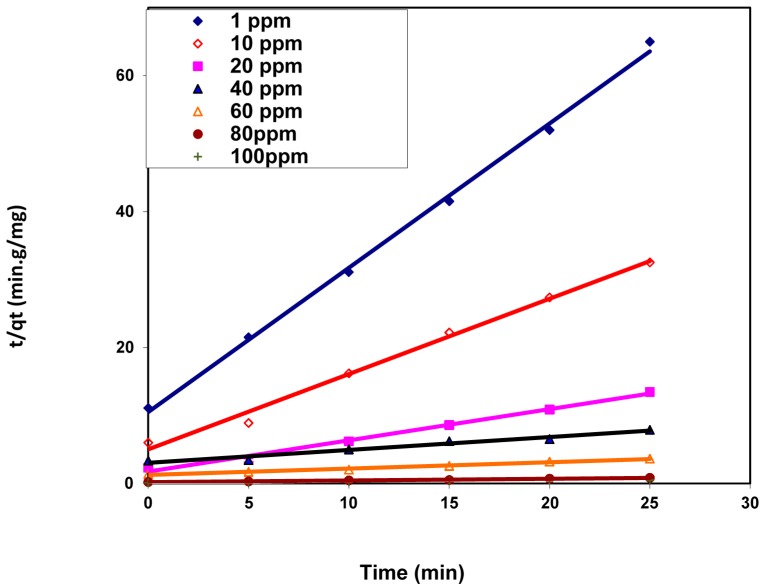
Pseudo-second-order kinetic model for BV 14 dye adsorption onto the chemically modified nanofibers.

It was indicated from Table 2 that the calculated theoretical equilibrium capacity values, *q*_e,cal_, are lower than that of the comparable experimental values, *q*_e,exp_, for the different studied dye concentrations. Moreover, the values of the correlation coefficients *R*^2^ of the model linear fitting are not extremely high. These results indicate that the dye adsorption process does not obey the pseudo-first-order kinetic model [23]. Blanchard *et al.* were the first developers of the pseudo-second-order model, and they based their assumption on the fact that the adsorption process obeys second-order chemisorption [24]. The linearity of this model is written as:

*t*/*q_t_* = (1/*k*^2^*q*_e_^2^) + *t*/*q*_e_ (7)

where *k*^2^ (g/mg·min) is the rate constant of the dye adsorption process. This rate constant and the theoretical dye capacity for each studied dye concentration were calculated from the linear plot of *t*/*q_t_* *vs.* *t* (Figure 9) and listed in Table 2.

This table shows that the correlation coefficient values *R*^2^ for the different studied dye concentrations are close to 1 and their values are larger than the comparable correlation coefficients derived from the pseudo-first-order kinetic model. Accordingly, the pseudo-second-order model can describe the dye adsorption process onto the nanofibers. These results suggested that the adsorption of the cationic BV 14 dye onto the chemically modified nanofibers may be chemisorption [22].

In order to determine the rate-limiting step of the BV 14 dye adsorption process onto the chemically modified nanofibers, the kinetic data was analyzed using the intraparticle diffusion model through the following equation [18]

**Figure 10:**
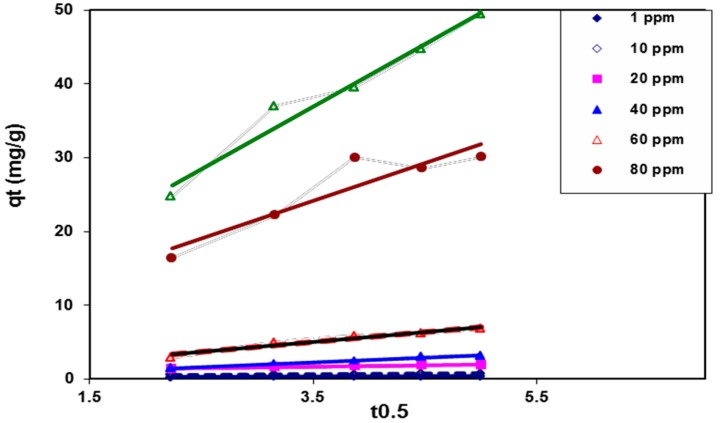
Intra-particle diffusion kinetic model for BV 14 dye adsorption onto the chemically modified nanofibers.

*q_t_* = *k*_id_*t*^1/2^ + *I* (8)

where *k*_id_ represents the intra-particle diffusion rate constant that relates to the boundary layer thickness. The plotting of the intraparticle diffusion model for the adsorption of BV 14 at different initial dye concentrations was illustrated in Figure 10. It was indicated that the plot of *q_t_ vs.* *t*^1/2^ for each studied dye concentration began initially as a curvature portion followed by a linear portion. This plot may be described as the bulk diffusion of dye particles being assigned to the initial curvature portion. However, the linear portion was attributed to the intraparticle diffusion. Neither the curvature portion nor the linear portion passed through the origin. These results indicate that the boundary layer resistance may control the dye sorption process. These results indicate that the intraparticle diffusion was not the only rate-limiting step and other processes may affect the dye adsorption process onto the fabricated nanofibers [25].

As an attempt to determine the actual rate-controlling step involved in the BV 14 adsorption process onto the fabricated nanofibers, the kinetic data were further analyzed using the Boyd kinetic model according to the following equation [26]:

*F* = 1 − (6/π^2^) exp(−*B_t_*) (9)

where *B_t_* represents the mathematical function of *F*, and *F* is the solute adsorbed fraction at various times *t*.

*F* = *q*/*q*_α_ (10)

where *q* is the amount adsorbed at time *t* (mg/g) and *q*_α_ represents the amount adsorbed at infinite time (in the present study, it is 30 min). Substituting the first equation, the kinetic model is expressed as

*B_t_* = −0.4978 − ln(1 − *q*/*q*_α_) (11)

**Figure 11:**
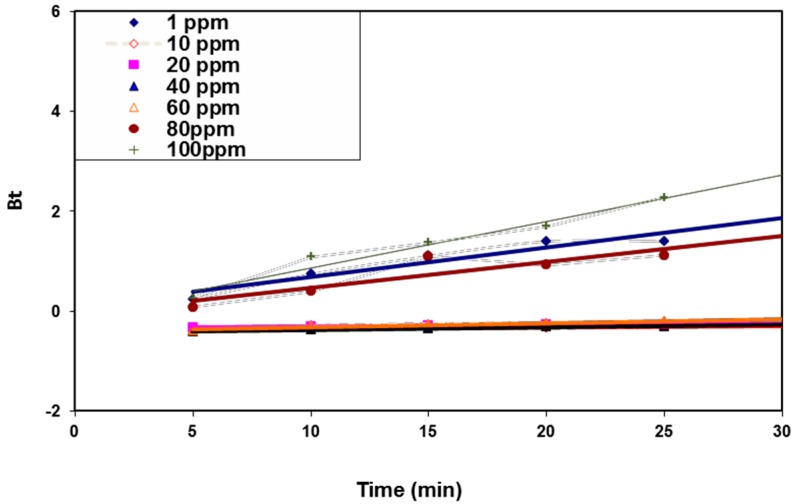
Boyd kinetic model for BV 14 dye adsorption onto the chemically modified nanofibers.

Figure 11 shows the so-called Boyd plots at different initial dye concentrations. The Boyd plots’ linearity was studied in order to determine whether the dye adsorption process was controlled by either the particle or by film diffusion. If the Boyd plot is a straight line passing through the origin, the particle diffusion mechanism predominates. However, for the plot that does not pass through the origin, the film diffusion or external mass transport mechanism predominates. Figure 10 shows that the plots were almost linear for all studied dye concentrations (average *R*^2^ ≥ 0.914) and did not pass through the origin. Accordingly, the film diffusion mainly governed the dye sorption process. The suggested adsorption mechanism may be due to the electrostatic interaction between the chemically modified nanofiber surface and the cationic dye molecules [27].

## 4. Conclusions

The functionalized fabricated poly(acrylonitrile-*co*-styrene) nanofiber demonstrated a good performance in the adsorption of basic violet dye (BV 14). The maximum dye adsorption capacity can reach 67.11 mg/g and the adsorption equilibrium was obtained in less than 30 min. The equilibrium dye sorption process was described using both the Langmuir and Temkin isotherm models. The adsorption of BV 14 onto the fabricated nanofiber is due to the chemisorption process. Moreover, the kinetics of the dye sorption process onto the nanofiber obeyed the pseudo-second-order model that confirms the chemisorption’s nature of the sorption process. The Boyd kinetic model for the dye sorption process showed that the film diffusion mainly governed the dye sorption process onto the fabricated nanofibers.

## Figures and Tables

**Table 1:** Estimated equilibrium parameters for the studied equilibrium isotherms of the dye adsorption process onto the chemically modified nanofiber.

Equilibrium isotherm	Parameters	Parameter values	Correlation coefficient fitting value (*R*^2^)
Langmuir isotherm	*q*_m_ (mg/g)	67.11	0.9954
	*K* (L/mg)	0.14	
	R_L_	0.877–0.0667	
Freundlich isotherm	*K*_F_ (mg/g)	2.8	0.866
	n_F_	1.1	
Temkin isotherm	*K_T_* (L/mg)	27	0.988
	*B* (kJ/mol)	7.1026	

**Table 2:** Estimated kinetic parameters for the different kinetic models of the dye adsorption process onto the chemically modified nanofibers.

Kinetic model	Parameter	Dye concentration						
		1	10	20	40	60	80	100
Pseudo-first-order	*K*_1_ (min^−1^)	0.07	0.087	0.099	0.11	0.1	0.1	0.1
	*q*_e,exp_ (mg/g)	0.45	4.66	9.3	18.9	28.1	37.65	46.65
	*q*_e,cal_ (mg/g)	0.2	4.5	7.4	14.7	22.7	31.7	42.2
	*R* ^2^	0.92	0.92	0.97	0.94	0.98	0.98	0.98
Pseudo-second-order	*K*_2_ (g/mg·min)	0.216	0.287	0.314	0.337	0.364	0.371	0.385
	*q*_e,exp_ (mg/g)	0.45	4.66	9.3	18.9	28.1	37.65	46.65
	*q*_e,cal_ (mg/g)	0.44	4.7	9.35	18.87	38.2	37.5	46.6
	*R* ^2^	0.997	0.99	0.998	0.998	0.998	0.997	0.99
Intra-particle Diffusion	*K*_d_ (mg/g·min^0.5^)	0.078	0.92	0.16	0.654	1.37	5.12	8.465
	*I* (mg/g)	0.89	0.92	1.09	0.03	0.23	6.26	7.36
	*R* ^2^	0.975	0.95	0.968	0.979	0.954	0.858	0.967
